# Supplementary material for: A Simple Method for Quantification of Polyhydroxybutyrate and Polylactic Acid Micro-Bioplastics in Soils by Evolved Gas Analysis
Source: Molecules. 2022 Mar 15;27(6):1898. doi: 10.3390/molecules27061898 (PMC8949261; doi:10.3390/molecules27061898)
Supplement: Supplementary file 1 [file molecules-27-01898-s001.zip › molecules-1599744-supplementary.pdf]

molecules

Supporting Information

A Simple Method for Quantification of Polyhydroxybutyrate and Polylactic Acid Micro-Bioplastics in Soils by Evolved Gas Analysis

Jakub Fojt [1,*], Ivana Románeková [1], Petra Procházková [1], Jan David [2], Martin Brtnický [1,3] and Jiří Kučerík [1]

[1] Institute of Chemistry and Technology of Environmental Protection, Faculty of Chemistry, Brno University of Technology, Purkynova 118, 612 00 Brno, Czech Republic; ivana.romanekova@vut.cz (I.R.); petra.prochazkova1@vut.cz (P.P.); martin.brtnicky@seznam.cz (M.B.); kucerik@fch.vut.cz (J.K.)
[2] Group of Environmental and Soil Chemistry, iES Landau, Institute for Environmental Sciences, University of Koblenz–Landau, Fortstraße 7, 76829 Landau in der Pfalz, Germany; david@uni-landau.de
[3] Department of Agrochemistry, Soil Science, Microbiology and Plant Nutrition, Faculty of AgriSciences, Mendel University in Brno, Zemedelska 3, 613 00 Brno, Czech Republic
* Correspondence: xcfojt@fch.vut.cz

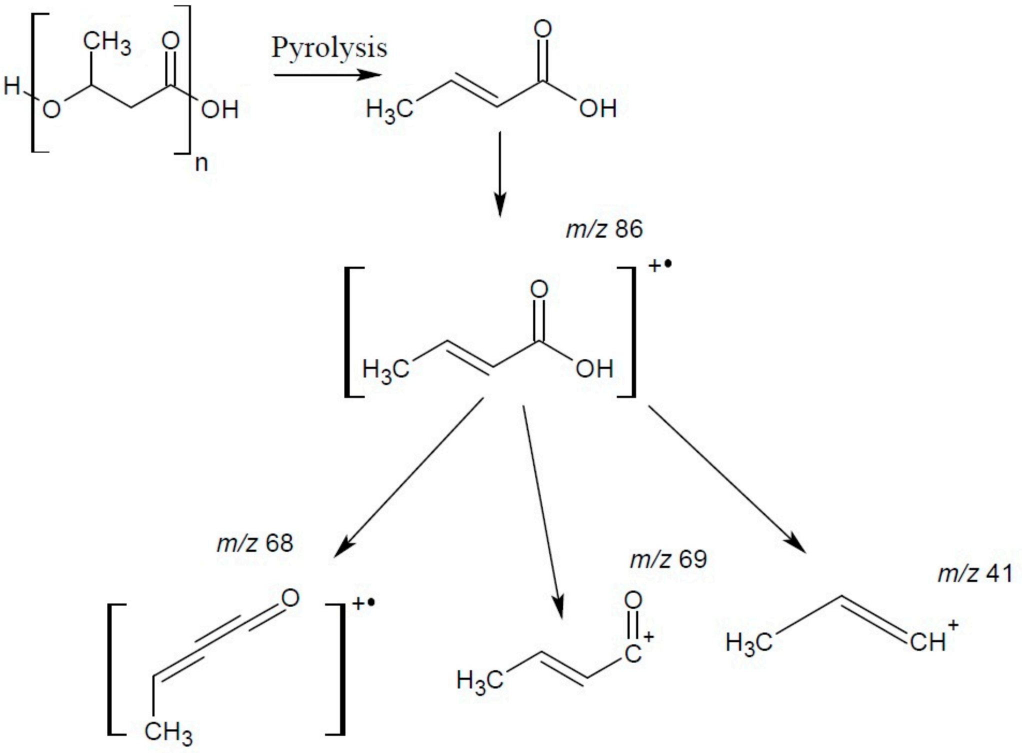

Figure S1. Degradation products and MS fragments of PHB.

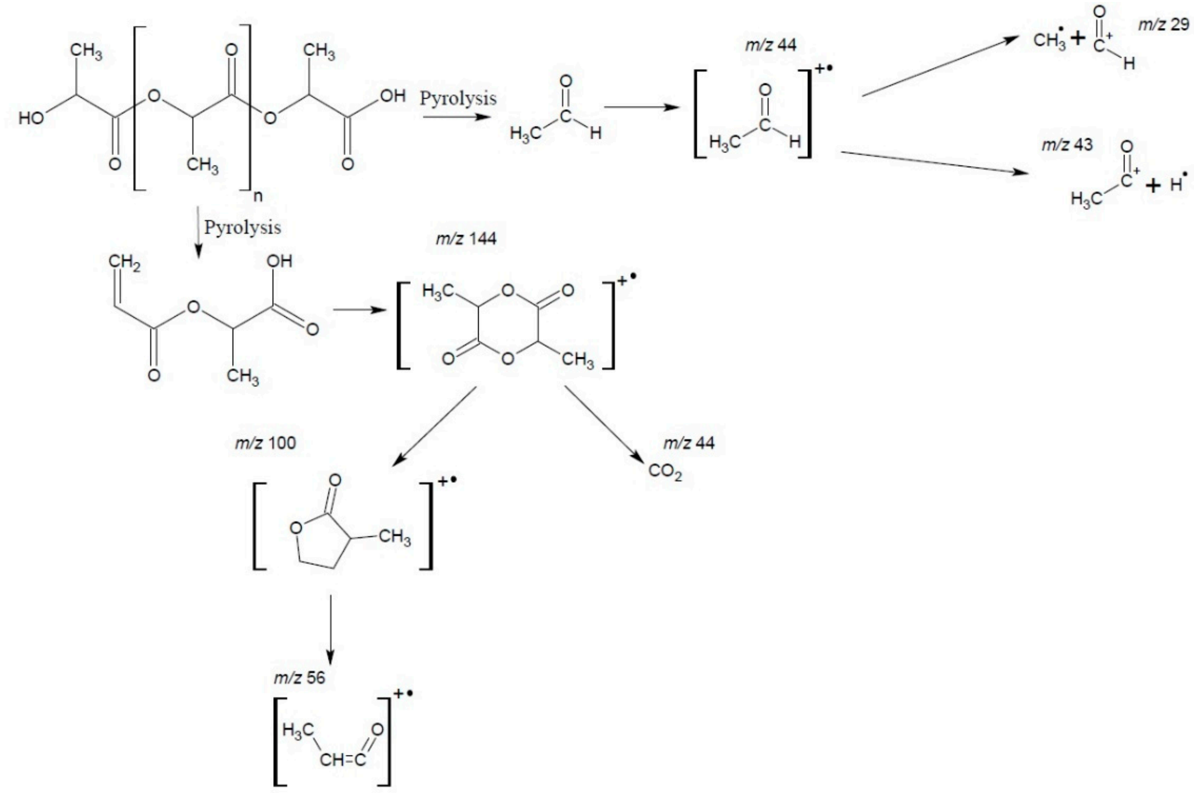

Figure S2. Degradation product and MS fragments of PLA.

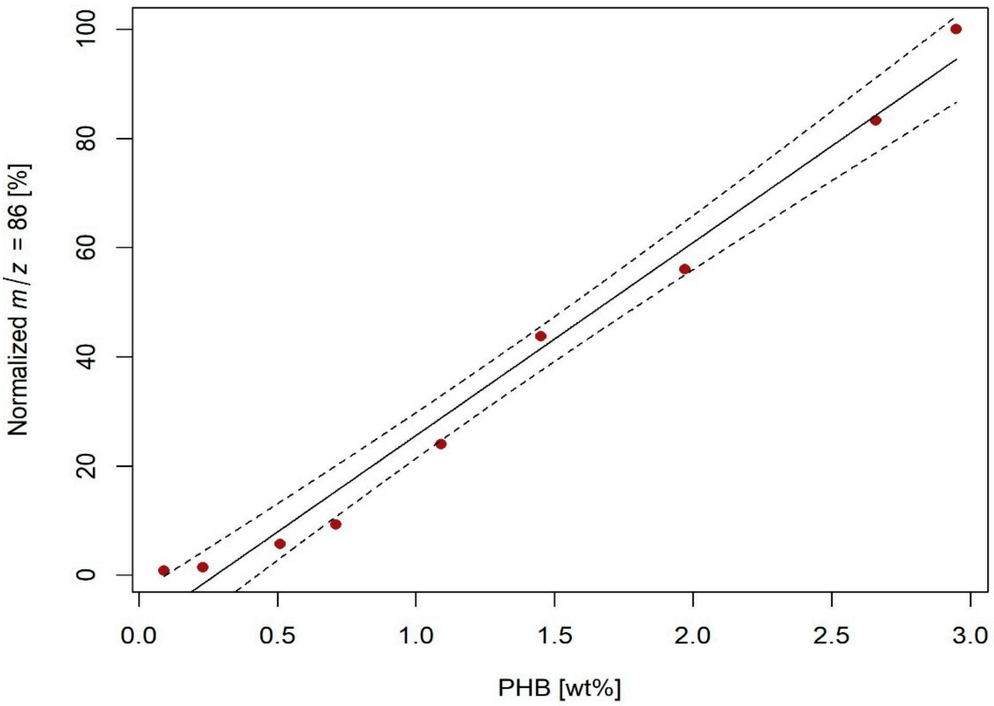

Figure S3. Calibration line for determination of PHB in HS5 soil by detection of degradation product with *m*/*z* 86, dashed line indicates 95% confidence interval.

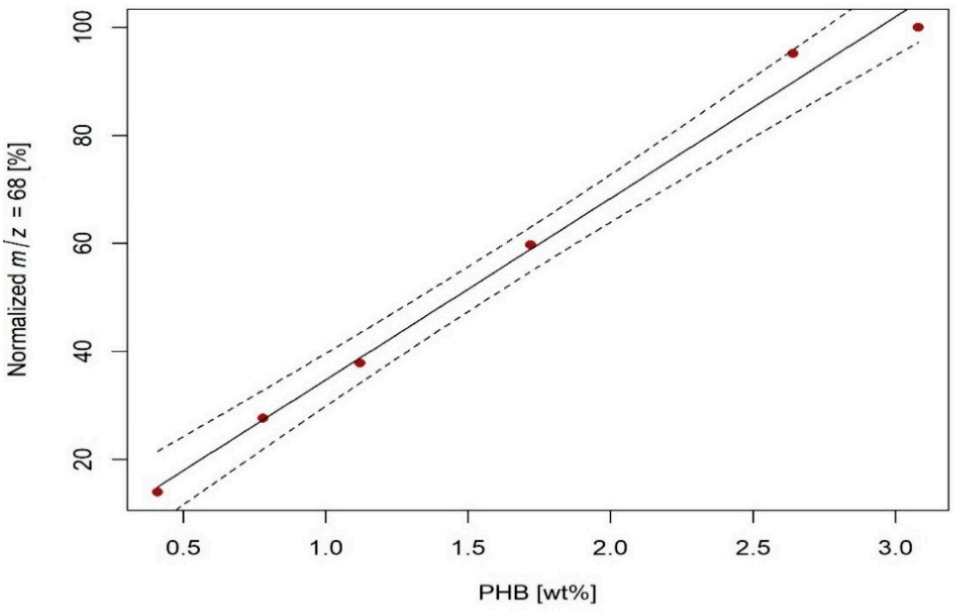

Figure S4. Calibration line for determination of PHB in HS45 soil by detection of degradation product with *m*/*z* 68, dashed line indicates 95% confidence interval.

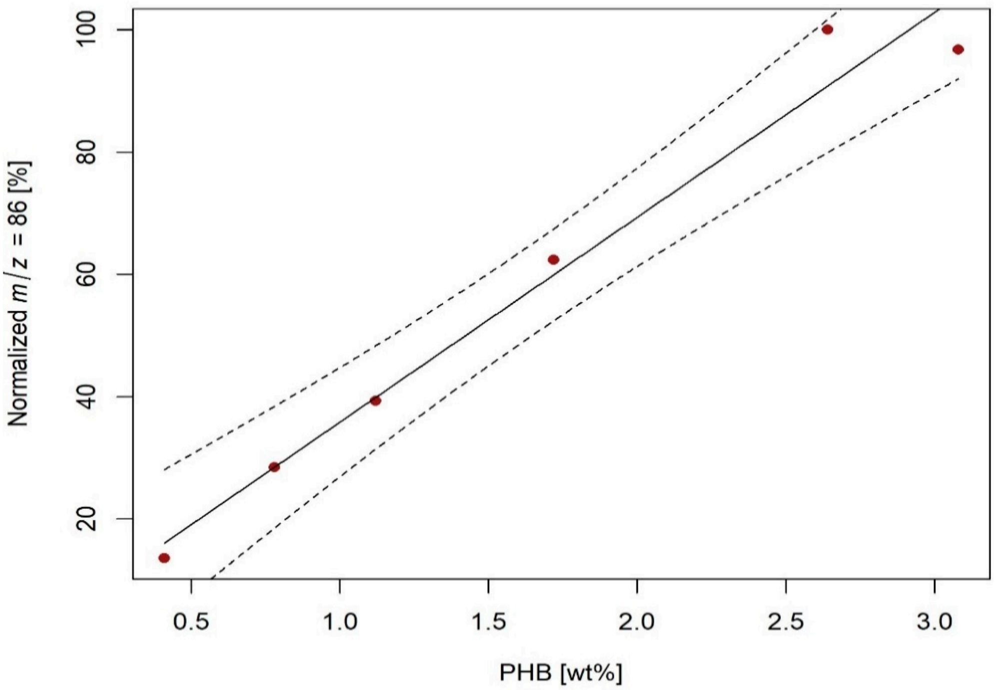

Figure S5. Calibration line for determination of PHB in HS45 soil by detection of degradation product with *m*/*z* 86, dashed line indicates 95% confidence interval.

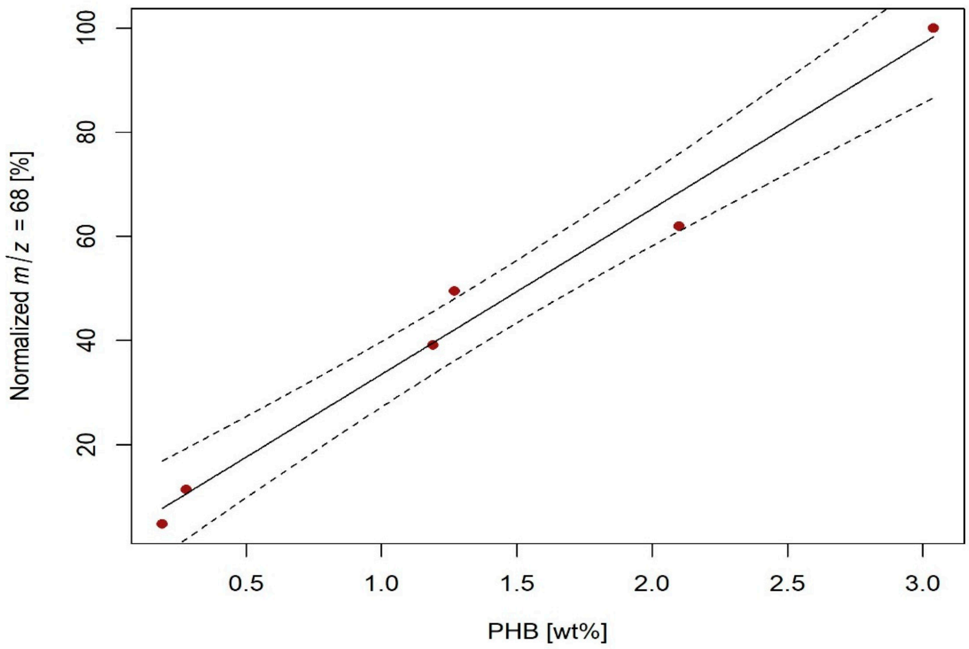

Figure S6. Calibration line for determination of PHB in LUFA soil by detection of degradation product with *m*/*z* 68, dashed line indicates 95% confidence interval.

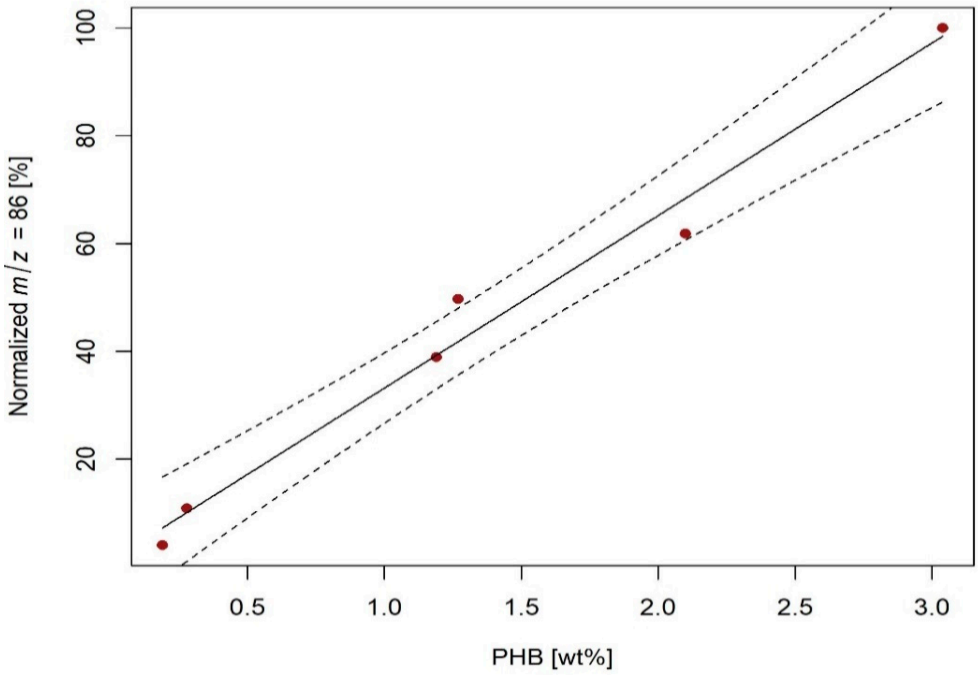

Figure S7. Calibration line for determination of PHB in LUFA soil by detection of degradation product with *m*/*z* 86, dashed line indicates 95% confidence interval.

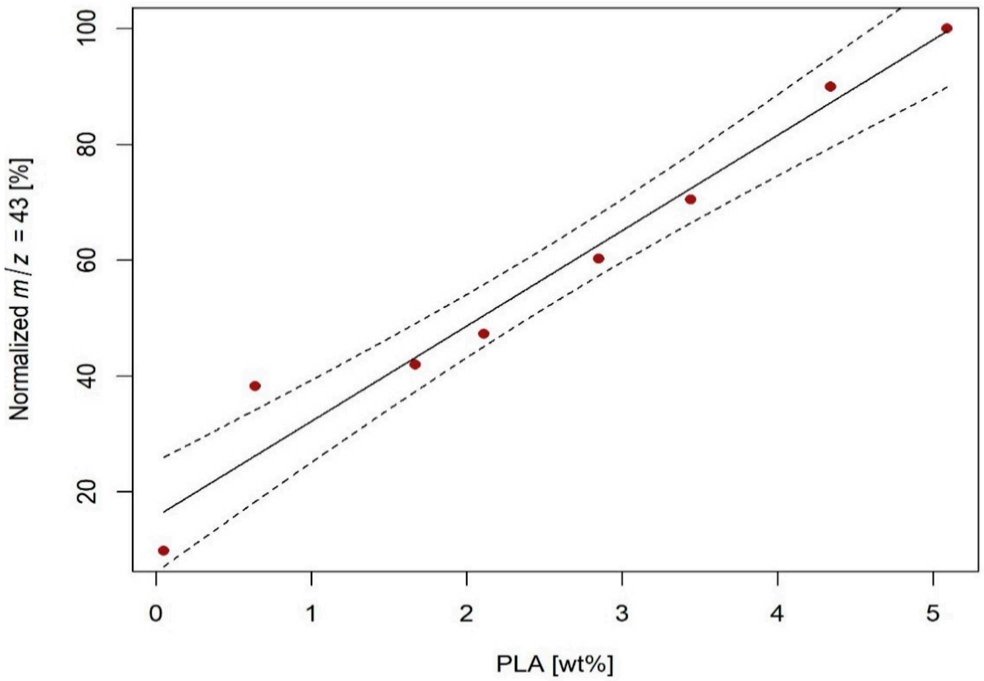

Figure S8. Calibration line for determination of PLA in P185 soil by detection of degradation product with *m*/*z* 43, dashed line indicates 95% confidence interval.

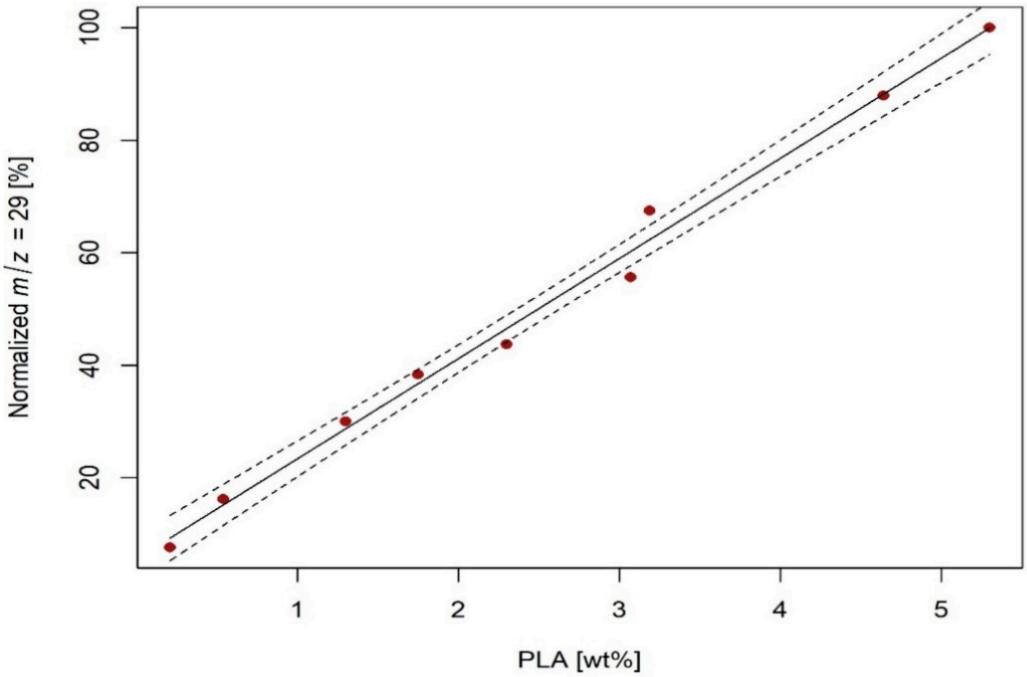

Figure S9. Calibration line for determination of PLA in P84 soil by detection of degradation product with *m*/*z* 29, dashed line indicates 95% confidence interval.

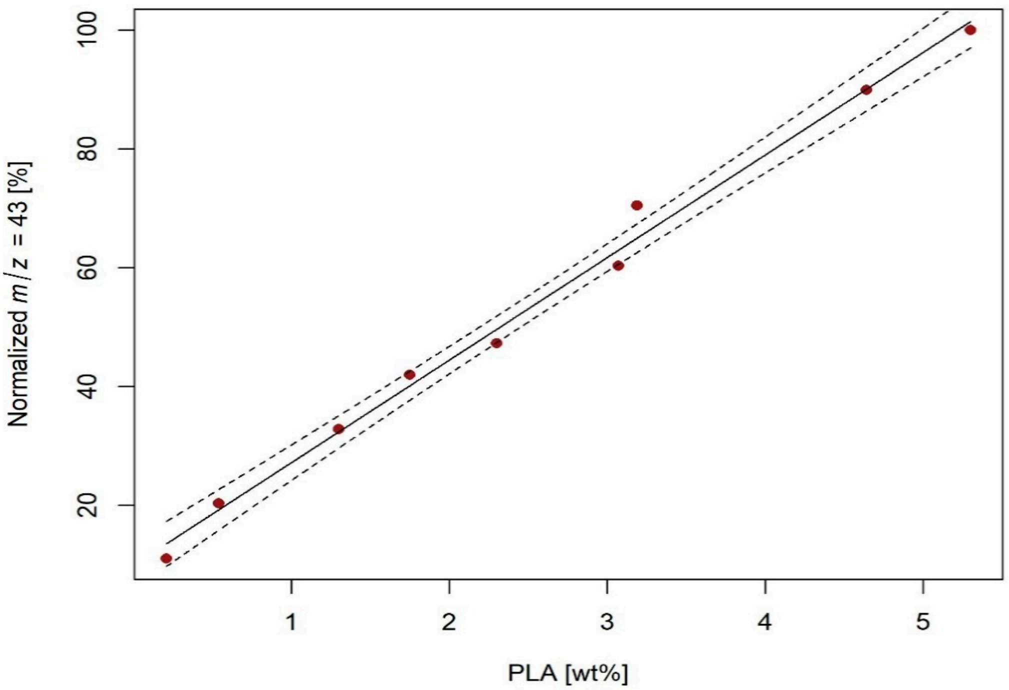

Figure S10. Calibration line for determination of PLA in P84 soil by detection of degradation product with *m*/*z* 43, dashed line indicates 95% confidence interval.

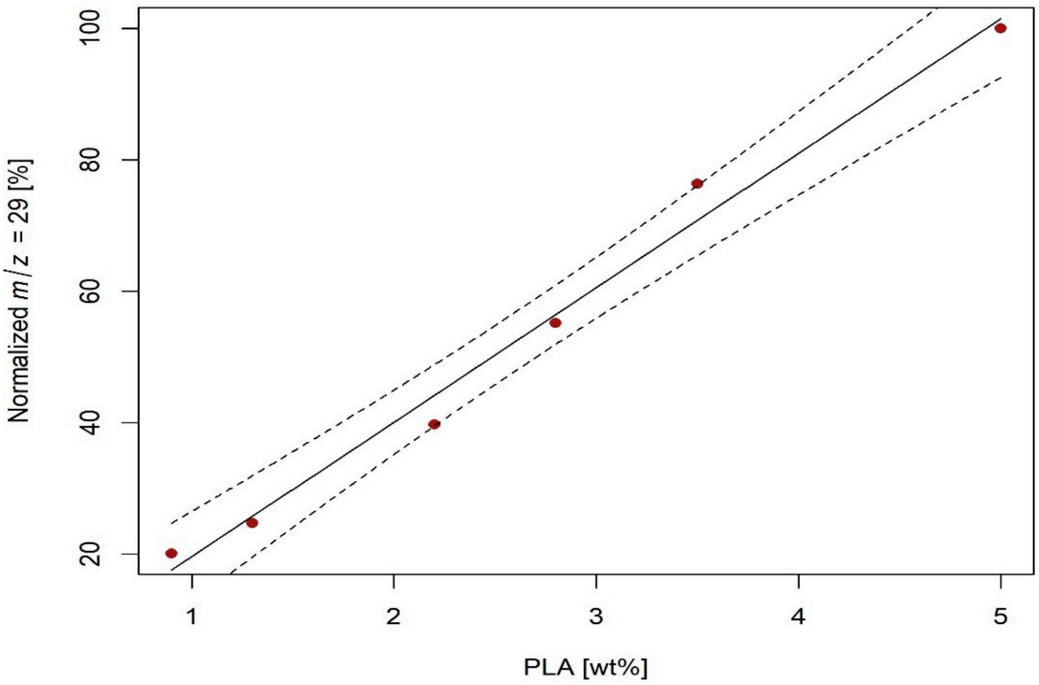

Figure S11. Calibration line for determination of PLA in LUFA soil by detection of degradation product with *m*/*z* 29, dashed line indicates 95% confidence interval.

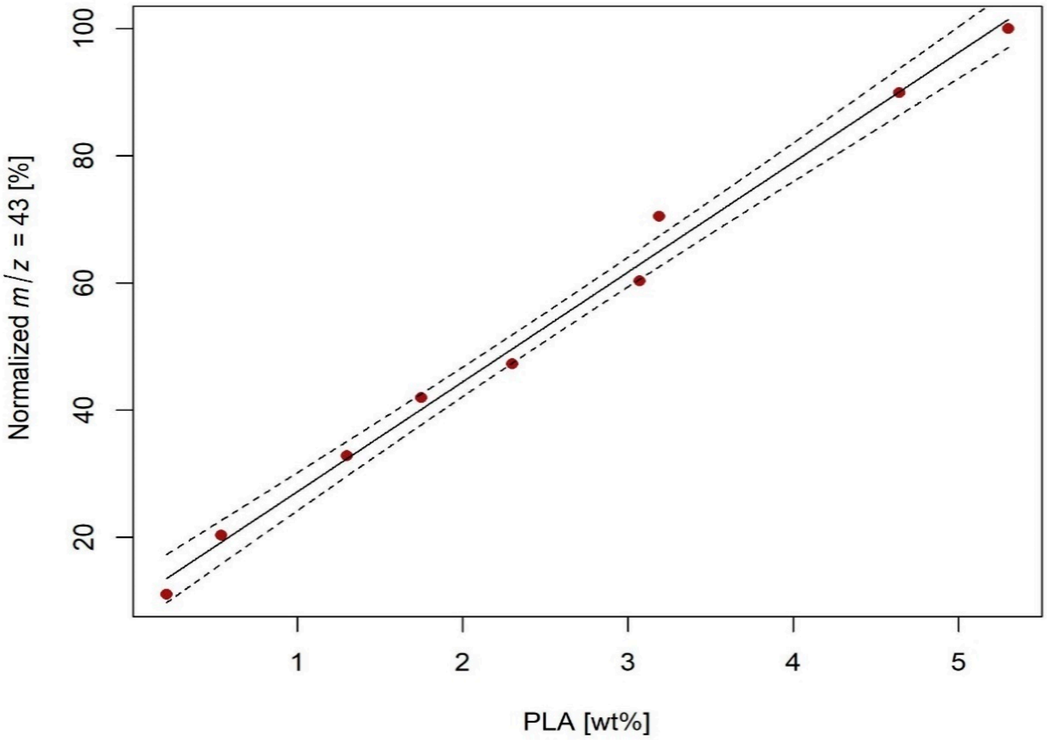

Figure S12. Calibration line for determination of PLA in LUFA soil by detection of degradation product with *m*/*z* 43, dashed line indicates 95% confidence interval.
